# Two-marker protein profile predicts poor prognosis in patients with early rectal cancer

**DOI:** 10.1038/sj.bjc.6604729

**Published:** 2008-11-04

**Authors:** I Zlobec, K Baker, L Terracciano, S Peter, L Degen, C Beglinger, A Lugli

**Affiliations:** 1 Institute of Pathology, University Hospital of Basel, Basel, Switzerland; 2 Department of Pathology, McGill University, Montreal, Canada; 3 Department of Gastroenterology, Brigham and Women's Hospital, Harvard Medical School, Boston, MA, USA; 4 Department of Gastroenterology and Hepatology, University of Alabama in Birmingham, Birmingham, AL, USA; 5 Department of Gastroenterology, University Hospital of Basel, Basel, Switzerland

**Keywords:** rectal cancer, prognosis, tumour marker, immunohistochemistry, receiver operating characteristic curve

## Abstract

The aim of this study was to establish an immunohistochemical protein profile to complement preoperative staging and identify rectal cancer patients at high-risk of adverse outcome. Immunohistochemistry was performed on a tissue microarray including 482 rectal cancers for APAF-1, EphB2, MST1, Ki67, p53, RHAMM, RKIP and CD8^+^ tumour infiltrating lymphocytes (TILs). After resampling of the data and multivariable analysis, the most reproducible markers were combined and prognosis evaluated as stratified by pT and pN status. In multivariable analysis, only positive RHAMM (*P*<0.001; HR=1.94 (1.44–2.61)) and loss of CD8^+^ TILs (*P*=0.006; HR=0.63 (0.45–0.88)) were independent prognostic factors. The 5-year cancer-specific survival rate for RHAMM+/TIL− patients was 30% (95% CI 21–40%) compared to 76% (95% CI: 66–84%) for RHAMM−/TIL+ patients (*P*<0.001). The 5-year cancer-specific survival of T1/T2/RHAMM+/TIL− patients was 48% (20–72%) and significantly worse compared to T3/T4/RHAMM−/TIL+ patients (71% 95% CI 56–82%); *P*=0.039). Stratifying by nodal status, only N+/RHAMM+/TIL− patients demonstrated a significantly worse prognosis than N0/RHAMM+/TIL− patients (*P*=0.005). Loss of CD8^+^ TILs was predictive of local recurrence in RHAMM+ tumours (*P*=0.009) only. RHAMM and CD8^+^ TILs may assist in identifying early stage rectal cancer patients facing a particularly poor prognosis and who may derive a benefit from preoperative therapy.

Preoperative radio- and chemoradiotherapy are now considered an integral part of treatment for patients with rectal cancer and can result in considerable tumour downstaging, downsizing or even complete pathological response in 20–30% of cases (Bosset *et al*, 2006; Mohiuddin *et al*, 2006). Even with total mesorectal excision (TME), neoadjuvant therapy continues to improve clinical outcome in patients with rectal cancer (den Dulk *et al*, 2008). The selection of patients for preoperative therapy is largely based on clinical staging made by endorectal ultrasound (EUS), computed tomography (CT) or magnetic resonance imaging (MRI). Biological markers predictive of poor clinical outcome from the preoperative biopsy would be useful tools to complement clinical staging. To date, such biological markers have had limited impact, including both the molecular analysis of *K-ras* and *p53*, as well as immunohistochemical markers (Turner *et al*, 2007; Guillem *et al*, 2008). There is currently no tissue-based marker, which is recommended as a prognostic factor by the European Group on Tumour Markers for patients with rectal cancer (Duffy *et al*, 2007). Possible reasons may include the difficulty in interpreting results from small studies, statistical analysis restricted to a single marker and the use of scoring methods for assessing immunoreactivity, which often may lack validation.

The aim of this study was to establish, using eight protein markers, an immunohistochemical protein profile, which can be applied in the preoperative setting to complement staging and help to identify patients with a high-risk of adverse outcome. The markers were selected for their representations of different cellular processes and for their established or potential prognostic value. In particular, apoptosis protease activating factor -1 (APAF-1) and mammalian sterile20-like kinase 1 are proapoptotic proteins, the reduced expression of these relate to adverse survival (Teraishi *et al*, 2006; Paik *et al*, 2007; Ren *et al*, 2008). Loss of Ephrin B2 receptor (EphB2) and the metastasis suppressor Raf-1 kinase inhibitor protein (RKIP) have been linked with poor outcome (Jubb *et al*, 2005; Al-Mulla *et al*, 2006), whereas expression of Ki67 and p53 have led to conflicting reports (Kyzer and Gordon, 1997; Munro *et al*, 2005). The receptor for hyaluronic acid-mediated motility (RHAMM) mediates both Ras and TGF-b signalling pathways and is associated with poor prognosis in a variety of tumour entities (Wang *et al*, 1998; Maxwell *et al*, 2004; Hamilton *et al*, 2007). Finally, CD8^+^ tumour infiltrating lymphocytes (TILs) have been linked to improved survival in colorectal cancer patients (Chiba *et al*, 2004; Galon *et al*, 2006). This study was carried out on 482 preoperatively untreated rectal tumours, using a reproducible scoring method, a systematic approach to determining negative or positive protein marker expression and validation of prognostic effects by resampling of the data.

## Materials and methods

### Patients

The patient collective was derived from three centres and included 1420 non-consecutive patients with primary colorectal cancer treated from 1987 to 1996 at the Institute of Pathology, University Hospital of Basel, Switzerland, the Institute of Clinical Pathology, Basel Switzerland and the Institute of Pathology, Stadtspital Triemli, Zürich, Switzerland.

### Tissue microarray (TMA) and immunohistochemistry

These 1420 colorectal cancers were retrospectively collected. Pathology was systematically reassessed for all cases. A TMA consisting of these tissues was constructed as described previously (Sauter *et al*, 2003). Clinicopathological data were available for all patients included on the TMA. The use of tissue for this study was approved by the local Ethics committee of the University Hospital of Basel.

Briefly, the 1420 colorectal cancers were dewaxed and rehydrated in dH_2_O. Antigen retrieval was performed using a pressure cooker in 0.001 M ethylenediaminetetraacetic acid (EDTA) pH 8.0. Endogenous peroxidase activity was blocked using 0.5% H_2_O_2,_ and the sections were incubated with 10% normal goat serum (Dako Cytomation, Carpinteria, CA, USA) for 20 min. Tissues were incubated with primary antibodies for APAF-1 (clone NCL APAF-1, Novocastra, Newcastle UK, 1 : 40), EphB2 (clone AF467, R&D Systems, Minneapolis, MN, USA, 1 : 200), Ki67 (clone MIB-1, Dako Cytomation, Glostrup, Denmark, 1 : 100), MST1 (polyclonal, Cell Signaling, Danvers, MA, USA, 1 : 200), p53 (clone DO-7, Dako Cytomation, Carpinteria, CA, USA, 1 : 200), RHAMM (clone 2D6, Novocastra, Newcastle, UK, 1 : 100), RKIP (polylonal, Upsatet, New York, NY, USA, 1 : 1000) and finally CD8^+^ TILs (clone C8144B, 1 : 100). Subsequently, sections were incubated with HRP-conjugated secondary antibody (DakoCytomation) for 30 min at room temperature. For visualisation of the antigen, the sections were immersed in 3-amino-9-ethylcarbazole+substrate-chromogen (DakoCytomation) for 30 min, and counterstained with Gill's haematoxylin.

Intraepithelial CD8^+^ TILs located in direct contact with tumour cells were quantified over the area of the entire punch for each case in the TMA. Evaluation of other immunohistochemical markers was performed semiquantitatively by assessing the proportion of immunoreactive tumour cells over the total number of tumour cells per TMA punch. A score ranging from 0 to 100% was ascribed to each tumour based on 5% intervals.

### Selection of rectal cancers and clinicopathological data

Tumours located in the colon (*N*=938) were excluded from the study. Analysis was restricted to carcinomas of the rectum and included 482 cases. All rectal cancers were preoperatively untreated. The clinicopathological features for these patients included gender, pT and pN stage, tumour grade, vascular invasion, mismatch-repair status and number of lymph nodes collected after resection (Table 1). The mean of age at diagnosis and of tumour diameter was 68.7 years (range: 36–96 years) and 46 mm (5–125 mm), respectively. Average number of lymph nodes collected was 11.8 (range: 0–61). For 90 patients, cause of death or status at the last follow-up was unknown and these patients were excluded from survival analysis. Survival time was therefore obtained for 392 patients. Follow-up ranged from 0 to 150 months with a median of 51 months. The 5-year cancer-specific survival time was 54% (95% confidence interval (CI): 49–59). Censored observations were defined as patients who were alive, lost to follow-up or suffered death from reasons other than rectal cancer up until 5-years following surgery. For 118 patients, information on the presence or absence of distant metastasis, local recurrence and postoperative therapy was available.

### Receiver operating curve (ROC) analysis

The cutoff scores for protein marker positivity were determined by ROC curve analysis (Zlobec and Lugli, 2008). This method can be used to determine the optimal cutoff points for protein marker expression when scored semiquantitatively, as was done in this study. At each expression score, the sensitivity and specificity for survival is determined and plotted, thereby generating an ROC curve. Using the (0, 1) criterion, the point on the curve that minimises the trade-off between sensitivity and specificity, thus having the shortest distance to the coordinate (0, 1), is chosen and patients are classified accordingly as positive or negative around this corresponding protein expression value. It should be noted that although prognosis is generally considered a time-to-event outcome, time-dependent ROC methods have only recently been established and to date can not be implemented with ease for the cutoff point determination over time (Heagerty *et al*, 2000). For this reason, standard ROC curve analysis was used in this study and is expected to yield the best cut off value for markers to discriminate between patients who have died from disease *vs* those alive or censored after 5 years.

### Statistical analysis

Univariate survival analysis using Cox proportional hazards regression was performed for each protein marker. The assumption of proportional hazards was verified before each analysis. Hazard ratios (HRs), 95% CI and *P*-values were used to determine the effect of each protein marker on survival time. In the case of protein markers, the baseline hazard of 1.0 was systematically attributed to negative protein expression. HR >1.0 indicate an adverse prognosis with positive expression, whereas HR <1.0 indicate improved prognosis with marker positivity. To determine the reliability of the prognostic effects of markers significant in univariate analysis, bootstrapped replications of the data were analysed. This approach allows one to sample the data with replacement, for example, 1000 times resulting in 1000 different ‘resamples’ of the original dataset. For each of these resamples, multiple Cox regression analysis was performed using a forward selection procedure. The number of times a particular marker was selected as an independent factor, after adjustment for the remaining variables, was determined. The most reliable independent markers were combined into multimarker phenotypes with different combinations of their negative or positive expressions. Kaplan–Meier survivals curves were analysed using the log-rank test. *χ*^2^-Tests were performed to determine the association of marker expression on the absence or presence of local recurrence. *P*-values were two-sided and considered statistically significant if <0.05. Analyses were performed using SAS (9.1, The SAS Institute, NC, USA).

## Results

### Survival analysis

#### Univariate analysis

The expression of four markers was associated with survival time including negative expression of Ki67 (*P*=0.033; HR=0.72 (0.53–0.97)), positivity for RHAMM (*P*<0.001; HR=2.19 (1.65–2.91)), absence of RKIP (*P*=0.015; HR=0.69 (0.51–0.93)) and loss of CD8^+^ TILs (*P*<0.001; HR=0.55 (0.41–0.74); Table 2).

### Reliability of prognostic markers

Ki67, RHAMM, RKIP and CD8^+^ TILs were entered into multivariable analysis with a selection procedure. For each of the 1000 resamples, only those markers, which remained significantly associated with survival time after adjustment for the prognostic effects of the remaining markers during forward selection (*P*<0.05) were selected. The most frequently selected markers were considered the most reliable prognostic factors. Out of the 1000 resamples, RHAMM, CD8^+^ TILs, Ki67 and RKIP were selected 990, 854, 244 and 69 times, respectively as prognostic factors.

### Multivariable analysis

The receptor for hyaluronic acid-mediated motility and CD8^+^ TILs, determined to be the most valuable markers, were entered into a second multivariable analysis along with clinicopathological features that are available at the time before surgery, namely pT stage, pN stage and age at diagnosis (Table 3). Positivity for RHAMM (*P*<0.001; HR=1.94 (1.44–2.61)) and loss of CD8^+^ TILs (*P*=0.006; HR=0.63 (0.45–0.88)) maintained their significant adverse effect on survival time.

### Two-marker protein profile

**Figure 1:**
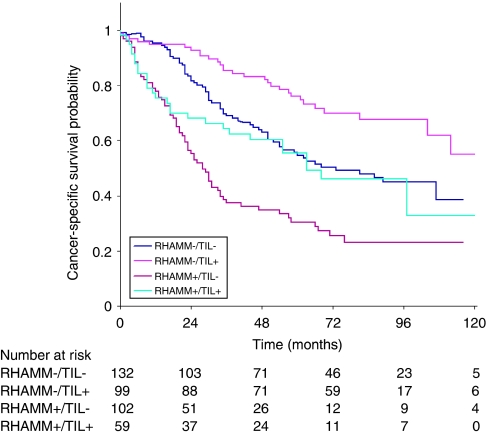
Kaplan–Meier survival curves and cancer-specific survival rates for combinations of RHAMM and CD8^+^ tumour infiltrating lymphocytes (TILs) (*P*<0.001).

The receptor for hyaluronic acid-mediated motility and CD8^+^ TILs were combined into their four possible phenotypes (RHAMM+/TIL+, RHAMM+/TIL−, RHAMM−/TIL+ and RHAMM−/TIL−). The Kaplan–Meier survival curve in Figure 1 highlights considerable differences between the four phenotypes with RHAMM+/TIL− tumours having significantly worsened (*P*<0.001) survival time compared to RHAMM−/TIL+ tumours. Moreover, the 5-year survival rate for patients with RHAMM+/TIL− tumours was 30% (95% CI: 21–40%) compared to 76% (95% CI: 66–84%) for RHAMM−/TIL+ patients.

**Figure 2:**
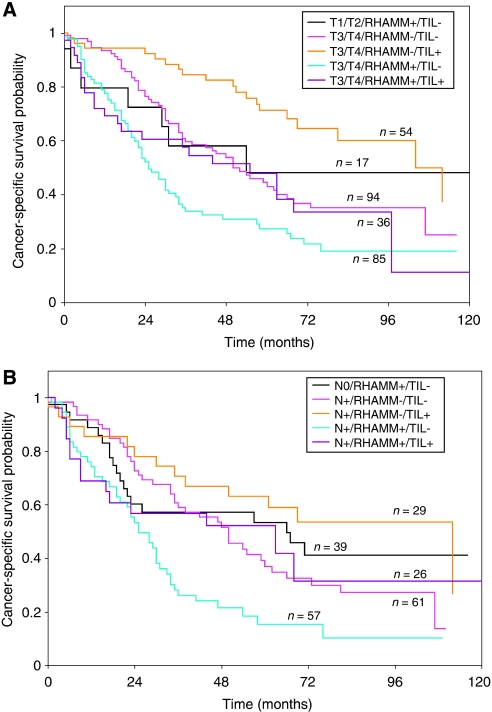
(**A**) Comparison of Kaplan–Meier survival curves and cancer-specific survival rates for early T1 and T2 patients with the highly adverse RHAMM+/TIL− phenotype compared with late T3 and T4 patients. (**B**) Comparison of Kaplan–Meier survival curves cancer-specific survival rates for node-negative (N0) patients with the highly adverse RHAMM+/TIL− phenotype compared with node-positive (N+) patients.

Figure 2A illustrates the survival time in patients with early T1, T2 tumours with the adverse RHAMM+/TIL− phenotype. The 5-year cancer-specific survival rate of these patients was 48% (95% CI: 20–72%), whereas for patients with the more favourable marker combination RHAMM−/TIL+, a 5-year survival rate of 84.4% (95% CI: 68–93%) was observed. Compared to the unfavourable early cancers, late T3, T4 patients with RHAMM−/TIL+ tumours had a significantly better prognosis (*P*=0.039) and 5-year survival rate of 71% (95% CI: 56–82%). Only late T3, T4 patients with RHAMM+/TIL− tumours performed significantly worse than patients with early T-stage tumours of adverse RHAMM+/TIL− phenotype.

Similar observations were made in Figure 2B for patients with node-negative (N0) RHAMM+/TIL− tumours compared with node-positive (N+) patients. Only N+ tumours with the RHAMM+/TIL− phenotype demonstrated a significantly worse prognosis (*P*=0.005), with 5-year cancer-specific survival rate of 15% (95% CI 6–28%), compared with N0 tumours with the adverse RHAMM+/TIL− phenotype (the 5-year survival rate: 53% (95% CI 35–69%)).

### Local recurrence

The receptor for hyaluronic acid-mediated motility and CD8^+^ TILs were tested for their association with local recurrence. In total, 87 patients with information on local recurrence were also evaluable for both markers of which 44.9% (*n*=39) had local failure. Neither RHAMM (*P*=0.606) nor CD8^+^ TILs (*P*=0.386) were predictive of this end point on their own. A highly significant statistical interaction (*P*=0.006) was observed between the two markers indicating that the effect of CD8^+^ TILs on local recurrence is modified by the negative or positive expression of RHAMM. These results are shown in Table 4. Loss of CD8^+^ TILs is significantly linked to the presence of local recurrence in RHAMM-positive tumours (*P*=0.009), a result which is not observed in RHAMM-negative patients.

## Discussion

Biological markers for identifying rectal cancer patients at high risk of poorer clinical outcome would improve the selection of patients for preoperative therapy. In this study on 482 rectal cancers, we document that combined negativity for CD8^+^ TILs and over-expression of the marker RHAMM leads to adverse survival independently of disease stage. Using a resampling technique, we determined that the prognostic effect of these two markers was remarkably reproducible in multivariable analysis. The novel findings of this study show that the combined immunohistochemical profile of RHAMM+/TIL− accurately predicts a highly adverse prognosis in patients with early stage disease. In particular, patients with T1, T2 tumours with this unfavourable protein profile have a 5-year cancer-specific survival rate similar to patients with T3 or T4 cancers. Similarly, N0 patients with RHAMM+/TIL− tumours were found to have comparable survival times to patients with N+ cancers. These findings indicate that immunohistochemical analysis of CD8^+^ TILs and RHAMM expression can not only refine prognostic subgroups in rectal cancer but could be utilised to identify a subgroup of early rectal cancer patients at high risk of adverse prognosis.

An understanding of the biological function of these two protein markers further clarifies their contribution to prognosis. Although few studies have specifically targeted the evaluation of CD8^+^ TILs in rectal cancer, several groups, including ours have previously identified intraepithelial TIL positivity as a favourable prognostic indicator in colorectal cancers, especially those characterised by microsatellite stability, a feature shared by the majority of rectal cancers (Baker *et al*, 2007). We have additionally reported the impact of absence of CD8^+^ TILs on local recurrence rates in mismatch repair-proficient colon cancer (Zlobec *et al*, 2008c). The close proximity of activated cytotoxic T cells to the tumour cells may allow for initiation of an effective antitumour immune response despite the generally immunosuppressive nature of the gut (Montufar-Solis *et al*, 2007). Furthermore, the presence of intraepithelial TILs likely reflects an active peripheral immune response capable of efficiently eradicating micrometastases and thereby contributing to prolonged patient survival. In this study, we find that the loss of CD8^+^ TILs is also linked to local recurrence in rectal tumours, but only in the context of RHAMM positivity.

The receptor for hyaluronic acid-mediated motility is a multifunctional glycoprotein often upregulated in advanced malignancies and has been identified as an adverse prognostic marker in both colorectal and breast cancers (Hamilton *et al*, 2007; Zlobec *et al*, 2008b). We have recently identified RHAMM in combination with p21 as highly conducive towards a severely adverse prognosis in microsatellite instability-high (MSI-H) colorectal cancer (Zlobec *et al*, 2008a). When expressed at the cell surface, RHAMM serves as a receptor for hyaluronic acid and has been implicated in both tumour cell motility and invasiveness (Hardwick *et al*, 1992). In N0 rectal cancers, RHAMM positivity may be indicative of a particularly invasive tumour phenotype prone to the release of micrometastases from the primary tumour. The dismal prognosis of patients with rectal cancers simultaneously negative for intraepithelial CD8^+^ TILs and overexpressing RHAMM may thus arise because of the failure of the host's immune system to contain an exceptionally motile type of cancer.

On the one hand, our study is limited by the fact that preoperative biopsies were not themselves analysed. Rather tissue punches from the central part of the tumour were used for immunohistochemistry. Our results, therefore, will require validation in a prospective setting. Additionally, survival time in patients with rectal cancer is known to be linked to surgical procedure. As our rectal cancer specimens were collected from 1987 to 1996, many of the patients included here predate the TME as the gold standard in care for advanced stage rectal cancer. This may explain to some degree the relatively higher frequency of local failure in our series. Interestingly, we found the rate of distant metastasis to be only 12%. We hypothesise that this may be related to the frequency of mismatch repair-deficient tumours in this study, which may be greater than the expected. Although little is known about the incidence of microsatellite instability specifically in rectal cancer, lower rates of distant metastasis have been observed in patients with this feature in colorectal cancer in general. In addition, because of the small number of people in our study treated in the adjuvant setting, we could not reliably test whether the prognostic effect of the marker combination RHAMM and CD8^+^ TILs is maintained after postoperative therapy. The poor prognosis in the subgroup of patients with RHAMM+/TIL− tumours is independent of pT or pN stage suggesting that this multimarker phenotype could be helpful in selecting patients for adjuvant chemotherapy.

Our study is strengthened by the TMA technique, which has allowed us to study eight different markers on 482 tumours. In conjunction with the described scoring method and ROC curve analysis, the use of TMAs has been shown to promote strong interobserver agreement between independent pathologists (Zlobec and Lugli, 2008). Moreover, all patients were preoperatively untreated in this series, and so no adjustments for the possible effects of neoadjuvant therapy were required.

This study strongly suggests that the two-marker immunohistochemical protein profile of RHAMM and CD8^+^ TILs can identify patients with adverse prognosis independent of the extent of disease. Added to the preoperatively available array of patient information, the combined analysis of RHAMM and CD8^+^ TILs could assist in selecting early stage rectal cancer patients most likely to be facing a particularly poor prognosis and who may derive the most benefit from preoperative therapy.

## Figures and Tables

**Table 1:** Clinicopathological features of rectal cancer patients

	**Frequency**		
**Clinicopathological feature**	**No. available**	**%**	**Total (*n*)**
*Sex*			
Male	251	52.1	482
Female	231	47.9	
			
*pT stage*			
pT1	29	6.1	475
pT2	112	23.6	
pT3	301	63.4	
pT4	33	7.0	
			
*pN stage*			
pN0	238	52.2	456
pN1	122	26.8	
pN2	96	21.1	
			
*Tumour grade*			
G1 and G2	443	93.5	474
G3	31	6.5	
			
*Vascular invasion*			
Absent	346	73.0	474
Presence	128	27.0	
			
*Invasive margin*			
Pushing	176	37.1	474
Infiltrating	298	62.9	
			
*Mismatch repair*			
Proficient	451	93.6	482
Deficient	31	6.4	
			
*Recurrence*			
Absent	62	53.9	115
Present	53	46.1	
			
*Metastasis*			
Absent	104	88.1	118
Present	14	11.9	
			
*Postoperative therapy*			
None	91	78.5	116
Radiotherapy	5	4.3	
Chemotherapy	11	9.5	
Radiochemotherapy	9	7.8	
			
*Survival*			
No. deaths	226	57.7%	392

**Table 2:** Association of protein marker expression and 5-year cancer-specific survival time in patients with rectal cancer (univariate analysis; Cox proportional hazards regression)

**Protein marker**	**Cutoff score**	**Negative *N* (%)**	**Positive *N* (%)**	**HR (95% CI)**	***P*-value**
APAF-1	90%	226 (53.2)	199 (46.8)	0.98 (0.74–1.29)	0.876
EphB2	70%	150 (39.8)	227 (60.2)	0.92 (0.66–1.27)	0.549
Ki67	15%	221 (57.6)	163 (42.5)	0.72 (0.53–0.97)	0.033
MST1	70%	124 (31.2)	274 (68.8)	0.76 (0.56–1.02)	0.068
p53	20%	199 (46.9)	225 (53.1)	1.08 (0.82–1.42)	0.584
RHAMM	90%	239 (59.3)	164 (40.7)	2.19 (1.65–2.91)	<0.001
RKIP	80%	128 (34.5)	243 (65.5)	0.69 (0.51–0.93)	0.015
CD8^+^ TILs	four cells/HPF	275 (60.0)	183 (40.0)	0.55 (0.41–0.74)	<0.001

HPF=high-power field; HR=hazard ratios; CI=confidence interval.

**Table 3:** Association of RHAMM and CD8^+^ TILs after adjusting for the prognostic effect of pT stage, pN stage, age and tumour diameter (multivariable analysis; Cox proportional hazards regression)

	***P*-value**	**HR (95% CI)**
pT stage	<0.001	2.52 (1.61–3.94)
pN stage	<0.001	1.98 (1.44–2.71)
Age	<0.001	1.03 (1.02–1.05)
RHAMM	<0.001	1.94 (1.44–2.61)
CD8^+^ TILs	0.006	0.63 (0.45–0.88)

HR=hazard ratio; CI=confidence interval.

**Table 4:** Interaction between RHAMM expression and CD8^+^ TIL and their effect on local recurrence

		**CD8^+^ TILs** ***No.* (%)**		
**RHAMM expression**	**Recurrence**	**Negative**	**Positive**	***P*-value**
Negative				
	Absent	18 (58.1)	13 (41.9)	0.228
	Present	17 (73.9)	6 (26.1)	
				
Positive				
	Absent	12 (70.6)	5 (29.4)	0.009
	Present	4 (25.0)	12 (75.0)	
